# Process Evaluation of the Community Pharmacist-Led Allergic Rhinitis Management (C-PhARM) Service in Singapore

**DOI:** 10.3390/pharmacy7020056

**Published:** 2019-06-07

**Authors:** Joanne Shi Ying Yap, Colin Wei Qiang Tang, Helena Mei Ling Hor, Joy Boon Ka Chong, Kai Zhen Yap

**Affiliations:** 1 Faculty of Science, Department of Pharmacy, National University of Singapore, Blk S4A Level 3, 18 Science Drive 4, Singapore 117543, Singapore; joanneyap94@gmail.com; 2 Department of Pharmacy, Watson’s Personal Care Stores Pte Ltd, Fuji Xerox Towers #10-00, 80 Anson Rd, Singapore 079907, Singapore; colin.tang@watsons.com.sg (C.W.Q.T.); helena.hor@watsons.com.sg (H.M.L.H.); joy.chong@watsons.com.sg (J.B.K.C.)

**Keywords:** allergic rhinitis, community pharmacy, pharmacy practice, self-care, Singapore

## Abstract

A community pharmacist-led allergic rhinitis management (C-PhARM) service involving structured patient assessment, individualised recommendations and follow-up was developed in Watson’s Personal Care Stores Pte Ltd (Singapore) to ensure optimal allergic rhinitis (AR) self-management and appropriate use of intranasal corticosteroids (INC) in Singapore. This retrospective study aimed to evaluate the C-PhARM service processes and identify areas for improving the quality of service. Relevant data was extracted from archived clinical forms, customer satisfaction surveys and pharmacist quality improvement surveys to evaluate the “reach”, “recruitment”, “context” and “fidelity” of service implementation, as well as the “intervention delivered” and “received”. Over the nine months since the launch of the C-PhARM service in April 2016, 45 customers were enrolled, and 32 (71.1%) customers had received at least one follow-up. Recommendations provided at baseline included oral antihistamines (32, 71.1%), INC sprays (28, 62.2%) and counselling on non-pharmacological strategies (27, 60.0%). Among the 29 customers who exited the service, 20 (69%) responded to a satisfaction survey. Although customers deemed pharmacists to be professional and knowledgeable in providing clear and detailed information about AR, pharmacists reported a lack of protected time and interest from customers as service barriers. Sufficient protected time is required for pharmacists to effectively provide clinical service in a community pharmacy.

## 1. Introduction

Allergic rhinitis (AR) is an immunoglobulin E (IgE)-mediated disorder triggered by allergen exposure [1,2] that affects up to 13.1% of Singapore’s population [3]. The main sensitising agent of this allergic airway disease in Singapore’s tropical urban environment is house dust mite [4]. As AR is characterised by nasal and ocular symptoms similar to the common cold [1,2], sufferers tend to treat the disease lightly. Self-medication is thus common behaviour, and a physician’s advice for treatment is seldom sought [5,6]. Although AR is not life-threatening, uncontrolled AR symptoms can have a significant impact on health-related quality of life, sleep and day-to-day productivity [7,8], particularly in Asian countries where perennial allergies and symptoms of AR are prevalent [9,10]. Therefore, the community pharmacists’ role in ensuring safe and effective self-management of AR has significant value in improving the quality of life.

In Singapore, AR self-management was found to be potentially suboptimal due to the underuse of INC and non-adherence to treatment [11]. To overcome this, a community pharmacist-led allergic rhinitis management (C-PhARM) service involving structured patient assessment, individualised treatment recommendations and follow-up was developed by the clinical team at Watson’s Personal Care Stores Pte Ltd and launched officially in April 2016 at all Watsons pharmacies [12].

## 2. Objectives 

While outcome evaluation is often conducted to determine the effectiveness of a health intervention program, process evaluation can provide a detailed understanding of the contributing elements and barriers to the success of a program and derive suggestions for optimising future intervention delivery [13,14,15]. Therefore, this study aimed to evaluate the processes of the C-PhARM service over the first nine months (April–December 2016) by adopting the methodology of Saunders and colleagues [14] and the Medical Research Council (MRC) [15]. Findings from this study will identify areas for improvement of the current C-PhARM service and provide useful insight for the development, implementation and evaluation of other clinical services in community pharmacy chains. 

## 3. Methodology

### 3.1. Study Design and Ethics Approval

This was a retrospective study using archived data from the C-PhARM service records dated April to December 2016. This study was approved by the Institutional Review Board of the National University of Singapore (NUS-IRB S-17-268).

### 3.2. Description of the C-PhARM Service and Implementation 

C-PhARM is an ongoing service for customers who seek pharmacists’ advice on AR at any Watsons pharmacy and consents to clinical follow-up from pharmacists. Individuals below the age of consent (21 years) are excluded. Customers who are pregnant, are nursing mothers, or have uncontrolled moderate–severe persistent AR, and symptoms not associated with AR or asthma (suspected undiagnosed or uncontrolled) are also excluded from the service as they should be referred to a physician for assessment and management. The exclusion criteria are in line with the 2008 Allergic Rhinitis and its Impact on Asthma (ARIA) guidelines [1] on which the C-PhARM pharmacist intervention protocol is based.

#### 3.2.1. Pharmacist Interventions

**Figure 1:**
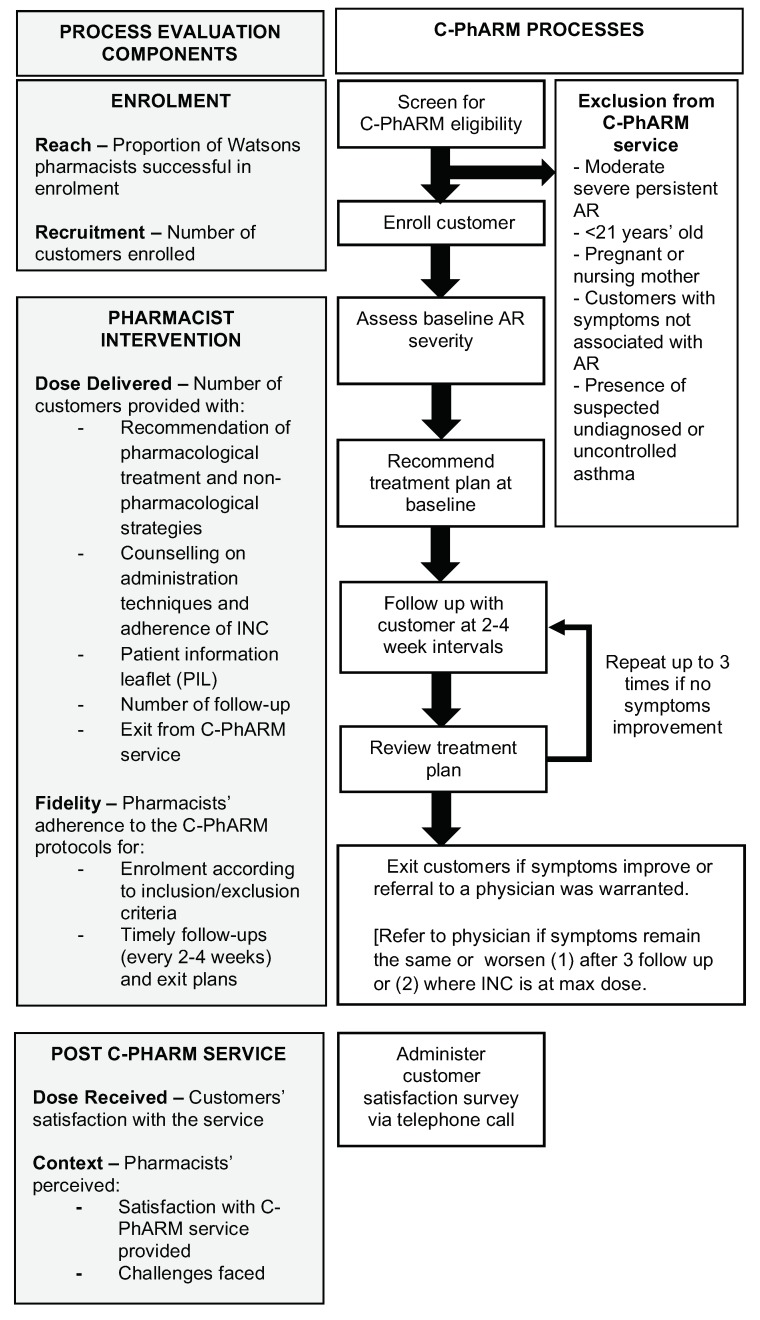
Flow of the community pharmacist-led allergic rhinitis management (C-PhARM) processes and evaluation component.

The C-PhARM pharmacist interventions are summarised in Figure 1.

##### Assessment and Recommendations at Baseline

After enrolment, customer-reported AR symptoms and duration were ascertained and classified by the attending pharmacist according to the ARIA severity classification [1]. This was then documented on the C-PhARM hardcopy baseline assessment form, along with the customer’s relevant demographic information, medical and medication history and pharmacist-recommended treatment plan, which may include the use of pharmacologicals such as antihistamines (with or without decongestants) and INC spray, along with counselling on the use of suitable non-pharmacological management strategies. A patient information leaflet (PIL) on AR and counselling on the administration techniques and adherence of INC were also provided to customers where appropriate. 

##### Assessment and Recommendations at Follow-Up

Telephone follow-ups with customers were provided by pharmacists every 2 to 4 weeks to assess treatment outcomes in terms of changes in AR symptoms and customers’ adherence to INC (if using). Adherence and continuation of treatment with INC are important to achieve the benefit of long-term reduction in symptoms, unlike oral antihistamines or decongestants that are to be used on a when needed basis. Hence, although all recommendations (including nonpharmacologic) were reviewed, adherence to INC was addressed specifically.

Based on the assessment at each follow-up, the attending pharmacist then reviewed the treatment plan and provided appropriate counselling to encourage adherence to INC and/or use of non-pharmacological management strategies where appropriate. The assessment and recommendations provided at follow-up were then documented on the hardcopy follow-up form. Where necessary, a maximum of three follow-ups was provided for each enrolled customer. 

##### Exit Plans and Protocol

Customers were exited from the service if an improvement in AR symptoms was observed or if they were referred to a physician (Figure 1). A satisfaction survey was then conducted via telephone calls within two weeks by members in the clinical C-PhARM team who were not involved in providing the C-PhARM service. Customers who were uncontactable after three calls on separate occasions were considered as dropouts from the C-PhARM service.

#### 3.2.2. Dissemination of the C-PhARM Protocol and Materials to Pharmacists

At the launch of the service, a C-PhARM kit comprised of (1) a pictogram of the workflow protocol for enrolment and follow-up process, (2) a clinical executive summary on the guidelines for patient assessment, management of AR, and pharmacotherapy recommendation, and (3) samples of the documentation forms were provided to all 60 pharmacists at 38 outlets. In addition, targeted individual face-to-face detailing of the materials in the C-PhARM kit was provided to 21 resident pharmacists at the 13 Watsons pharmacies that had the top INC sales from October to November 2015. All pharmacists’ queries regarding the C-PhARM service were also collated and addressed via email. 

### 3.3. Definitions of the Process Evaluation Components and Outcome Measures

Based on the MRC framework, the process evaluation components included in this study were “reach”, “recruitment”, “fidelity”, “dose delivered” and “context” [15]. In addition, the process evaluation framework proposed by Saunders and colleagues [14] was also adopted to include the “dose received” by customers as the sixth evaluation component. The outcome measures and data extracted from the C-PhARM service notes for each of these components are summarised in Figure 1. 

#### 3.3.1. Reach and Recruitment

Reach refers to the proportion of the target population who were offered an intervention, which indicates a program’s extent of reaching its intended audience [14,15]. As it was not feasible to track the percentage of AR patients who were offered C-PhARM in a retrospective study, the proportion of Watsons pharmacists who were successful in customer enrolment was used as the proxy outcome measure to reflect reach. In addition, the change in pharmacist participation before and after the detailing process was also assessed. Recruitment was reported as the number of AR customers enrolled in the C-PhARM service.

#### 3.3.2. Dose Delivered

Dose delivered refers to the amount of intervention delivered [14,15]. This was reported as the number of C-PhARM pharmacist interventions provided, which included pharmacological and non-pharmacological recommendations, provision of PIL, INC administration techniques and adherence counselling, the number of follow-ups, and exit plans. 

#### 3.3.3. Fidelity

Fidelity measures the consistency of the implemented intervention with what was planned [14]. Outcome measures included the proportion of enrolment, follow-ups, and exit plans that conform to the C-PhARM protocol.

#### 3.3.4. Dose Received

Dose received refers to a participant’s satisfaction with the program and their interaction with the pharmacist who provided the intervention [14]. In this study, customer-reported satisfaction with customers’ increased understanding of AR and related medication use, the frequency of follow-up received, and the professionalism and knowledge of pharmacists in providing clear and detailed information about the patient’s AR condition were measured. In addition, the extent to which customers found the PIL provided to be a good reference and whether they would recommend the C-PhARM service to their friends were also assessed. 

#### 3.3.5. Context

Context includes the external factors (barriers and facilitators to implementation) that could influence the effectiveness of the intervention in a program [14,15]. Specific to this study, pharmacist-reported perception of the overall C-PhARM service, the pharmacists’ level of motivation in customer enrolment, as well as the clarity and usefulness of the C-PhARM kit, the effectiveness of the one-on-one detailing process, and the challenges encountered were evaluated.

### 3.4. Data Collection

Data pertaining to customer demographics, as well as pharmacist-provided assessment and recommendations (pharmacological and non-pharmacological) at baseline and follow-up(s), were extracted from the archived C-PhARM hardcopy baseline assessment and follow-up forms for data analysis of C-PhARM’s recruitment, reach, intervention fidelity and dose delivered. Data for evaluating the dose of the intervention received by customers were obtained from the responses to the customer satisfaction survey that were archived in the C-PhARM service database.

Data for evaluating the context of C-PhARM was obtained from the pharmacists’ responses to a quality improvement survey that was administered by the C-PhARM clinical team to all 56 Watsons’ pharmacists (excluding the clinical team members involved in the development of the C-PhARM service and materials) in December 2016. Responses were captured on hardcopy self-administered questionnaires distributed during the Watsons pharmacy practice meeting and online questionnaires from pharmacists who were absent from the meeting. Both the customer satisfaction survey and quality improvement survey for pharmacists were developed based on the opinions of the study team. 

### 3.5. Data Analysis

All data were analysed using Microsoft Office Excel 2013 and reported using descriptive statistics in terms of frequencies, percentages and medians with interquartile ranges (IQR).

## 4. Results

### 4.1. Reach and Recruitment

A total of 45 customers were enrolled in the C-PhARM service by 13 (23.2%) pharmacists of Watsons between April and December 2016. The total number of pharmacists providing the service increased from 12 to 13 after detailing was provided. As shown in Table 1, the enrolled customers were predominantly Chinese (88.9%), female (60.0%), aged 31 or over (62.2%) and had a minimum tertiary-level education (82.2%).

### 4.2. Dose Delivered

#### 4.2.1. Pharmacist Interventions at Baseline

At the baseline consultation, antihistamine was the most commonly recommended treatment (n = 32, 71.1%). Counselling on non-pharmacological AR management strategies was provided for 27 (60.0%) customers. Of these, allergen avoidance (n = 18) and the use of normal saline wash (n = 13) were the most commonly discussed. 

PIL was provided to five (11.1%) customers while three (6.7%) customers explicitly rejected the PIL; no documentation of this intervention was found for the rest. Of all 28 customers who were recommended INC, INC adherence and/or administration technique counselling were provided for 17 (37.8%) customers. The details of the pharmacists’ interventions are summarised in Table 2.

#### 4.2.2. Pharmacist Interventions at Follow-Up and Exit Plans

**Figure 2:**
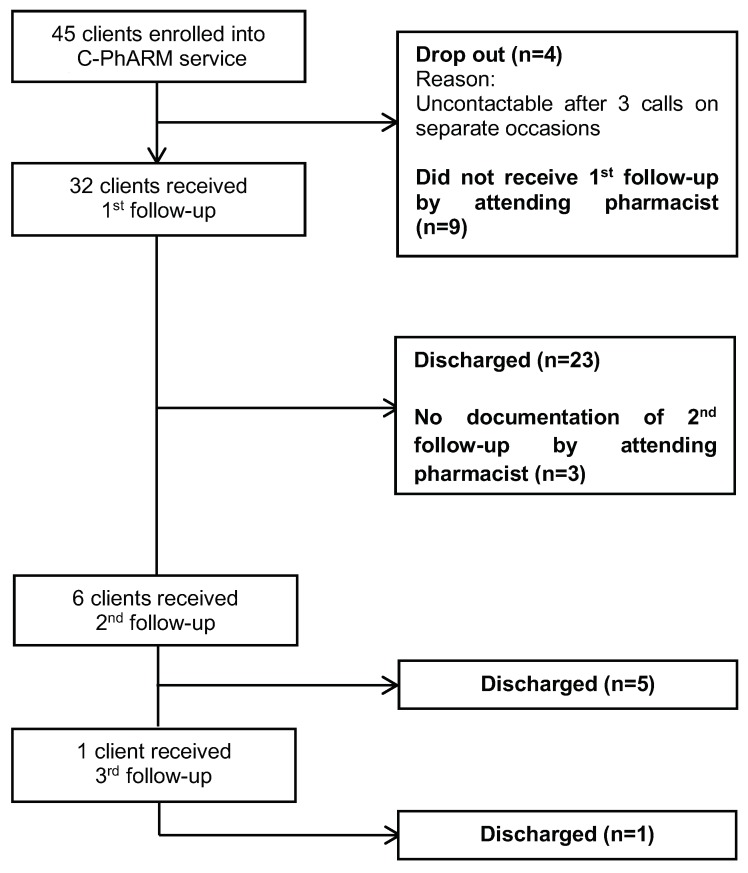
Flowchart of customers enrolled, followed up, and exited from the C-PhARM service.

Among the 45 customers enrolled, four were uncontactable and were considered to have dropped out of the service before the first follow-up. In all, follow-ups were provided for 32 (71.1%) customers, among whom six (13.3%) received at least two follow-ups. Subsequently, 29 (64.4%) customers were exited from the C-PhARM service (Figure 2). However, no documentation of follow-ups was found for nine customers. 

### 4.3. Fidelity

#### 4.3.1. Enrolment 

Among the 45 customers enrolled, 33 (73.3%) fulfilled the enrolment criteria whereas 12 (26.7%) were identified by attending pharmacists to have moderate–severe persistent AR but were not excluded (Table 2). Of these 12 customers, eight (17.8%) were followed up, among whom seven (15.6%) eventually exited the service after showing improvement, and one was referred to a physician.

#### 4.3.2. Follow-Up and Exit Plans

Among all 32 first follow-ups provided to the 45 enrolled customers, 29 (64.4%) were made within 2–4 weeks from the initial assessment. All seven (15.6%) subsequent follow-ups to six customers were made every 2–4 weeks from the previous ones. Overall, the average duration between follow-ups was 21 ± 9.6 days. 

A total of 29 (64.4%) customers exited from the service in accordance with the C-PhARM exit guide. Among them, 23 (51.1%) reported symptom improvement, two reported being satisfied with the outcomes despite no symptom improvement, and four (8.9%) were referred to a doctor for further assessment.

### 4.4. Dose Received (Customer Satisfaction)

Among the 29 customers who exited from the C-PhARM service, 20 (response rate = 69.0%) replied to the customer satisfaction survey; two customers were uncontactable for the survey, and seven refrained from participating. Overall, customers responded positively to all the items (Table 3). Particularly, the attending pharmacists were deemed to be professional and knowledgeable in providing clear and detailed information about AR (median = 4, IQR 4–5). The customers were also comfortable with the frequency of follow-up and expressed that C-PhARM improved their overall experience with community pharmacy (median = 4, IQR 4–4.75).

### 4.5. Context

#### 4.5.1. Pharmacist Perceptions about the C-PhARM Service and Use of Guides

A total of 43 pharmacists responded to the C-PhARM quality improvement survey, providing a response rate of 76.8%. In general, pharmacists responded positively (with median ratings of 4) to most of the questions pertaining to their perception of the service, materials and detailing process (Table 4). However, pharmacists reported being somewhat ambivalent (median = 3) about the usefulness of the PIL and following the C-PhARM guides as compared to exercising professional judgement when giving treatment recommendations for customers. 

#### 4.5.2. Barriers to the C-PhARM Service

Among the challenges listed in the survey (Table 4), the need for protected time (median = 4, IQR 4–4), the lack of interest by customers to participate (median = 4, IQR 4–4) and the reluctance of customers to fill in the baseline assessment form (median = 4, IQR 3–4) were identified as the major challenges faced during customer enrolment, initial AR assessment and treatment recommendations. The major challenge that pharmacists faced during customer follow-up was the difficulty in conducting phone follow-ups without being interrupted by customers from the shop floor (median = 4, IQR 3–4). 

It was encouraging to note that pharmacists found the C-PhARM service to be beneficial in helping customers manage AR and were motivated to enrol patients (median = 4, IQR 3–4). In addition, pharmacists did not find the customer follow-up process to be confusing (median = 2, IQR 2–3).

## 5. Discussion

This study identified areas where the C-PhARM service can be improved. Firstly, although customers with moderate–severe persistent AR should be referred to a physician (as stated in the C-PhARM exclusion criteria), it was found that these customers preferred consulting with the pharmacists for self-management, especially when their symptoms had been officially diagnosed as AR before. Under such circumstance, it would be appropriate to continue following up with the customer to ensure the efficacy and safety of self-management rather than rejecting them from the C-PhARM service [1]. Thus, the C-PhARM enrolment criteria could be expanded to benefit such customers with moderate–severe persistent AR who were previously diagnosed by a physician as they can benefit from pharmacists’ counselling on appropriate and effective self-medication (e.g. INC administration technique and adherence).

Next, the lack of protected time could have contributed to the lapse in the timely follow-ups reported. Dispensaries are often managed by only one community pharmacist who has to juggle many responsibilities ranging from professional activities, such as dispensing of medications and patient counselling, to logistical activities for ensuring an adequate supply of products [16]. Compared to telephone calls (which will require the full presence and attention of the pharmacist for the duration), the use of other communication technologies such as emails/chat messages [17] and digital mobile health applications for follow-up can be a more convenient option for both pharmacists and customers. As replies through such communication channels do not need to be immediate, follow-up processes may suffer less from disruptions by urgent demands at the shop floor. However, emails and messages can be easily ignored or forgotten, potentially resulting in low response rates. 

In addition, documenting the interventions can be a time consuming and demanding process [18]. Therefore, the lack of protected time could have contributed to the lack of documentation, hence resulting in the low reported numbers of counselling on INC administration technique and adherence (17 customers out of 28 who were initiated with INC). However, documentation is an important process indicator that is essential for evaluating the quality of clinical pharmacist service [19]. More can be done to improve the user-friendliness of the C-PhARM service forms and/or emphasise the importance of this process. 

It is not apparent why PIL was provided to only five (11.1%) customers. Besides the possible reason of the lack in documentation, other contributing reasons cannot be ruled out, which may include the unavailability of PIL at the stores as they were not delivered in time or the rejection by customers as they were not literate in English. 

Lastly, the pharmacist-reported lack of customer interest in the service (coupled with the lack of protected time) could have resulted in the poor pharmacist participation rate. The lack of interest from customers may be due to their unfamiliarity with the benefits of pharmacy services or as a result of AR trivialisation. As suggested in a 2005 study conducted in Singapore by Chui and Li [20], there is a lack of consumer awareness that community pharmacists can help them self-medicate more safely and effectively. Perhaps, advertising the C-PhARM service to emphasise its benefits may help reach the consumers and improve public awareness. 

This study has several limitations. Firstly, this study focused only on the quantitative evaluation of the C-PhARM processes. Future studies should be conducted to evaluate patient outcomes using validated symptom scoring tools so as to gather more insight into the effectiveness of the C-PhARM service. Future qualitative studies can also be conducted to gain insight into the implementation processes and provide triangulation of data to better understand what actually transpires during service provision, to derive more targeted solutions to improve the quality of the service [21]. Secondly, the reported number of interventions delivered was only of those that were explicitly documented by pharmacists. Hence, the interventions reported could be underestimated. Thirdly, patient satisfaction is key to the successful management of AR patients [22] and is an important and commonly used indicator for measuring quality in healthcare [23]. Although customer feedback about C-PhARM was generally positive, the response rate to this survey was only 70.0% of those who exited, and 44.4% of all customers enrolled. Thus, non-response bias could be present, and the findings may not be representative of all customers enrolled in the C-PhARM service. Lastly, due to the retrospective nature of this study, the total number of AR customers who sought a pharmacist’s advice at Watsons pharmacy could not be determined. Therefore, we were unable to compute the reach (percentage of target customers being offered C-PhARM) or the rate of customer enrolment in the C-PhARM service. Although reach could be derived by tracking the number of individuals who purchased AR medication, this would not yield an accurate estimate as AR medication could also be used for other purposes, such as symptom relief of respiratory tract infections. Although recruitment (n = 45) was low given the study period, it was also not possible to determine whether reach was low. Albeit, the number of pharmacists who were successful in enrolling customers, reported as a proxy measure for reach, was also low (n = 13, 23.2% of all pharmacists). 

Moving forward, to improve the reach and recruitment, since pharmacists may often be busy at the dispensary, pharmacy assistants assisting customers with over-the-counter (OTC) medicines can be trained to assist with triage, assess customers who purchase OTC AR medications, and refer them for the pharmacist’s intervention under the C-PhARM service. In addition, workflow efficiency can also be improved by integrating the C-PhARM clinical documentation to the dispensing system. Although the C-PhARM protocol was based on the 2008 ARIA guidelines, they are aligned in principle to the recommendations in terms of pharmacotherapy and referrals described in recently published ARIA Pharmacy 2018 [24]. Although antihistamine and corticosteroid combination nasal sprays are currently not available for counter-prescribing by pharmacists in Singapore, there will be a need to review the C-PhARM protocol and materials with reference to the 2016 ARIA guidelines [25]—which discuss the place of therapy of intranasal antihistamines and corticosteroid combination products —when these products are reclassified and become increasingly accessible to the public.

## 6. Conclusions

In conclusion, this process evaluation provides insight into the contributing factors and barriers to the effective implementation of the C-PhARM service. Results indicate that community pharmacists should be provided with sufficient protected time and an environment free of interruption from customers to effectively provide quality clinical intervention and conduct follow-ups by more convenient means. The procedure used in this study may also inform the design of future process evaluations of related pharmacist-led services. 

## Figures and Tables

**Table 1:** Demographics of customers enrolled in the community pharmacist-led allergic rhinitis management (C-PhARM) service (n = 45).

Customer Profile	n	(%)
Gender		
Male	18	40.0
Female	27	60.0
Age		
21–30	17	37.8
31–40	11	24.4
41–50	9	20.0
51–60	6	13.3
>60	2	4.4
Ethnicity		
Chinese	40	88.9
Malay	3	6.7
Indian	0	0.0
Other	2	4.4
Education level		
Primary school and below	1	2.2
Secondary school	7	15.6
Polytechnic/ Junior college/ Institute of Technical Education	7	15.6
University and above	30	66.7
Level of physical activity		
Less than 150 min/week	28	62.2
About 150 min/week	9	20.0
More than 150 min/week	8	17.8
Smoking status		
Yes	1	2.2
Ex-smoker	4	8.9
No	40	88.9
Associated comorbidities		
Asthma only	9	20.0
Eczema only	10	22.2
Both asthma and eczema	2	4.4
None	24	53.3

**Table 2:** Interventions delivered by pharmacists (n = 45).

Pharmacist Intervention	Number of Interventions Provided, n	(%)
**BASELINE**		
Assessment of AR		
Mild intermittent	10	22.2
Mild persistent	8	17.8
Moderate—severe intermittent	15	33.3
Moderate—severe persistent	12	26.7
Recommendations/Interventions		
First-generation antihistamine ± decongestant only	2	4.4
Second-/third-generation antihistamine ± decongestant only	13	28.9
INC only	11	24.4
INC + antihistamine (any)	17	37.8
Non-pharmacological strategies^†^	27	60.0
Counselling on INC administration technique and adherence^‡^	17	37.8
Provision of AR PIL	5	11.1
**FOLLOW-UP**		
Number of follow-ups		
1	26	57.8
≥ 2	6	13.3
Interventions at first follow-up		
Dose adjustment	6	13.3
Maintain current regimen	13	28.9
Discontinue current regimen	9	20.0
INC adherence counselling	2	4.4
Referral to a doctor	4	8.9
Non-pharmacological strategies^†^	13	28.9
Interventions at second/third follow-up		
Dose adjustment	0	
Maintain current regimen	3	6.7
Discontinue current regimen	1	2.2
INC adherence counselling	0	
Referral to a doctor	0	
Non-pharmacological strategies^†^	1	2.2
Exits plans^§^	29	64.4

AR: allergic rhinitis; INC: intranasal corticosteroid; PIL: patient information leaflet; ^†^Non-pharmacological strategies included one or more of the following: nasal saline wash, allergen avoidance, air purifier. ^‡^INC administration technique and adherence counselling was taken to be provided only if explicitly documented by the attending pharmacist on the intervention form. ^§^Cases were exited if a referral to a doctor for further assessment was made or if customers reported symptom improvement and/or satisfaction with their outcomes.

**Table 3:** Customer-reported satisfaction with the C-PhARM service (n = 20).

Survey Item	Median (IQR)^†^
I have a better understanding of AR and related medication use through this service.	4 (3.25–4.75)
Watsons’ pharmacists are professional and knowledgeable in providing clear and detailed information about my condition	4 (4.00–5.00)
I am comfortable with the frequency of follow-up.	4 (4.00–4.75)
The PIL is a good reference for me.^‡^	4 (3.00–5.00)
This program improves my overall experience with community pharmacy.	4 (4.00–4.75)
I would recommend this allergic rhinitis service to my friend.	4 (3.25–4.75)

AR: allergic rhinitis; PIL: patient information leaflet; ^†^All items were rated using the 5-point Likert scale: 1 = strongly disagree, 2 = disagree, 3 = neutral, 4 = agree, 5 = strongly disagree. ^‡^Responses to this item were based on only nine customers who received the PIL.

**Table 4:** Pharmacist-reported perception of the C-PhARM service.

Survey Item	Median (IQR)^†^
**Overall C-PhARM service**	**(N = 43)**
The C-PhARM service workflow is clear.	4 (3–4)
The hardcopy C-PhARM service forms are user-friendly.	4 (3–4)
The C-PhARM service is beneficial in helping patients manage their AR condition.	4 (3–4)
The Watsons AR PIL is useful in assisting me during patient education.	3 (3–3)
I am motivated to recruit patients.	4 (3–4)
Overall, I am satisfied with the C-PhARM service.	4 (3–4)
**C-PhARM in-house protocols and guidelines**	**(N = 43)**
The workflow protocol for patient enrolment and follow-up process is useful.	4 (4–4)
The clinical executive summary for patient assessment and management is useful.	4 (4–4)
The INC recommendation guidelines are useful.	4 (4–4)
The antihistamine recommendation guidelines are useful.	4 (3–4)
I prefer to use my own discretion when providing treatment recommendations.	3 (3–4)
Overall, I am satisfied with the materials in the C-PhARM kit.	4 (4–4)
**Individual one-on-one detailing at the store^‡^**	**(N = 17)**
The above is effective in helping me better understand the workflow.	4 (4–4)
The above is effective in answering my queries about the C-PhARM service.	4 (3–4)
Overall, I am satisfied with the above.	4 (4–4)
**Challenges faced by pharmacists**	**(N = 43)**
I find the patient enrolment process confusing.	3 (2–3)
I am too busy to enrol patients in the C-PhARM service.	3 (3–4)
I need one-on-one dedicated, undisturbed time at baseline consultation	4 (4–4)
Patient is not interested in participating.	4 (4–4)
Patient is reluctant to fill in baseline assessment form.	4 (3–4)
I am unsure of how to approach patients.	3 (2–3)
I do not see the need to enrol patients in the C-PhARM service as there is no value in the service to optimise a patient’s AR condition.	2 (2–3)
I find the patient follow-up process confusing.	2 (2–3)
I am too busy to follow up with patients.	3 (3–4)
I find it difficult to conduct phone follow-ups with patients without being interrupted by patients from the shop floor.	4 (3–4)
I do not see the need to follow up patients as AR can be easily self-managed.	3 (2–3)

AR: allergic rhinitis; PIL: patient information leaflet; ^†^All items were rated using the 5-point Likert scale: 1 = strongly disagree, 2 = disagree, 3 = neutral, 4 = agree, 5 = strongly disagree. ^‡^Responses to this section were based on 17 pharmacists who indicated that they received individual face-to-face detailing the process at the store.
